# Boredom–understanding the emotion and its impact on our lives: an African perspective

**DOI:** 10.3389/fsoc.2023.1213190

**Published:** 2023-06-29

**Authors:** David M. Ndetei, Pascalyne Nyamai, Victoria Mutiso

**Affiliations:** ^1^Africa Mental Health Research and Training Foundation, Nairobi, Kenya; ^2^Department of Psychiatry, University of Nairobi, Nairobi, Kenya; ^3^World Psychiatric Association Collaborating Centre for Research and Training, Nairobi, Kenya

**Keywords:** boredom, psychiatric aspects, African context, emotion, impact

## Summary

Boredom is a universal experience overlooked in the scientific community despite most people having experienced it at some point in their lives. Despite being a common emotion, boredom has received little attention compared to other emotions such as happiness or anger (Westgate and Steidle, 2020). Boredom is often considered a trivial and inconsequential emotion, but recent studies have shown that it can have a significant impact on our wellbeing, productivity, and even our health. This paper aims to explore the concept of boredom, its impact on lives and its psychiatric aspect.

## Defining boredom

Boredom is a state of mind characterized by a lack of interest, stimulation, or challenge. It is a subjective experience that can manifest in a variety of ways, including restlessness, apathy, and disinterest. Boredom can be caused by a lack of external stimulation or by internal factors such as a lack of motivation or a sense of purpose. It can arise from routine tasks, repetitive activities, or lack of novelty, which can result in a sense of time dragging or feeling stuck in a monotonous routine. Boredom can also arise from unmet expectations or a discrepancy between our desires and our current reality (Eastwood et al., 2012; Van Tilburg and Igou, 2017; Raffaelli et al., 2018).

## The impact of boredom

Boredom can occur in different settings, including work, school, relationships, and leisure time, and is more prevalent among men, youths, the unmarried, and those of lower income (Chin et al., 2017; Weybright et al., 2020). While boredom is often seen as an unpleasant experience, it can have both negative and positive effects on individuals. On the one hand, boredom can be a source of creativity and innovation in that when bored, brains are more likely to wander and explore new ideas or perspectives. Boredom can encourage one to seek novel experiences, discover new interests, or challenge oneself to learn and grow. For instance during the COVID-19 pandemic, boredom as a result of lockdowns and isolations helped many explore new ideas and discover new interests. Some explored content creation as well as other interests such as painting, cooking, baking and knitting (Morse et al., 2021). Boredom can also prompt to reflect on values, goals, and aspirations, and motivate to make changes in lives. In this sense, boredom can be seen as an opportunity for self-awareness and self-improvement. Studies have shown that people who experience moderate levels of boredom are more likely to engage in creative thinking and problem-solving (Elpidorou, 2018; van Tilburg and Igou, 2019).

On the other hand, boredom can also have negative consequences such as decreased productivity, poor mental health, and even physical health problems. In one study a significant percentage of participants−67% of men and 25% of women—preferred to administer electric shocks to themselves rather than experience boredom while sitting alone with their thoughts (Wilson et al., 2014). This finding highlights how much people generally dislike being bored. Boredom has also been associated with various negative behaviors, such as self-harm, substance use, and engaging in distractions like watching movies during work (Havermans et al., 2015; Weybright et al., 2015; Nederkoorn et al., 2016). To illustrate how boredom can lead to distraction at work, an air traffic controller was caught watching a crime thriller while at work during a quiet overnight shift instead of monitoring the space, exposing the controller's distraction as a result of boredom at work (Lowy and Henry, 2016). Similarly, a security guard had to call the police to release him from handcuffs after he handcuffed himself out of boredom and lost the key (Darrah, 2019). These incidents highlight how boredom can affect workers in critical jobs that require prolonged vigilance. Despite technological advances that have made their tasks safer and easier, boredom can still creep in and impact their performance (Cummings et al., 2016).

## Psychiatric aspects of boredom

Boredom has significant psychological and psychiatric aspects. It is not just a feeling of being uninterested or disengaged rather it can affect various aspects of mental health, cognition, and behavior. Research has shown that boredom is not only linked to depression, but it may also be both a risk factor and a symptom of depression (Sommers and Vodanovich, 2000; Goldberg et al., 2011; Eastwood et al., 2012; Mercer-Lynn et al., 2013; Spaeth et al., 2015). A study on 722 students found that students who scored high on boredom scale also scored high on depression scale an indication that boredom can be a psychological feature of depressed states (Spaeth et al., 2015). In another study among 823 undergraduate students aimed at exploring the relationship between boredom and depression, the correlation between boredom and depression was substantially high (*r* = 0.72, *p* < 0.001; Goldberg et al., 2011). Boredom can disrupt motivation, reduce pleasure, and interfere with goal-directed behavior, which can contribute to the development of depressive symptoms. Boredom is also associated with anxiety by triggering anxious thoughts and worries or exacerbate symptoms of already existing anxiety disorders (LePera, 2011).

Further, boredom is implicated in the development and maintenance of substance use disorders. When individuals feel bored, they may turn to substances, such as alcohol or drugs, as a way to cope with or alleviate their boredom (Weybright et al., 2015; Biolcati et al., 2016). Boredom can also increase impulsivity, leading individuals to engage in risky or sensation-seeking activities as a way to alleviate their boredom (Lee et al., 2007; Mercer-Lynn et al., 2013). In fact, a study on binge drinking behavior in adolescents found that boredom proneness was a significant predictor of binge drinking (Biolcati et al., 2016). This impulsive behavior can further exacerbate mental health issues, such as anxiety and depression (LePera, 2011).

Individual and cultural differences affect how boredom is experienced (Vodanovich et al., 2011). For instance, age and stage in life can influence how boredom is experienced and expressed. Children and adolescents may be more prone to boredom due to their developing cognitive abilities and limited autonomy while older adults, on the other hand, may experience boredom as a result of retirement or decreased social interaction (Martin et al., 2006).

## African context

In the African context, boredom takes on unique dimensions shaped by cultural norms, values, and social structures. African societies are characterized by their rich cultural heritage, diversity, and communal way of life (Abungu, 2011; Columbus, 2014). African cultures have a strong emphasis on community and social cohesion, and people often find meaning and fulfillment through social relationships, extended family networks, and collective activities (Columbus, 2014). For example, in some African cultures, communal activities such as storytelling, music, dance, and traditional ceremonies provide a rich source of stimulation, creativity, and social interaction, which may mitigate feelings of boredom (Idang, 2015; Guide, Guide). However, with the rapid modernization in many parts of Africa, there are increasing challenges to traditional ways of life thus boredom has become a prominent issue. This is more so in the wake of COVID-19 pandemic that limited socialization.

Another aspect of boredom in the African context is the impact of poverty and inequality. Many African countries face significant challenges in terms of poverty, unemployment, and limited access to basic resources and services (Ukpere, 2011; Francis and Webster, 2019). In such contexts, people may experience boredom due to a lack of opportunities for education, employment, and personal growth. The feeling of being stuck in a monotonous routine without prospects for improvement can lead to a sense of hopelessness.

Boredom has emerged as a significant mental health concern in the context of the COVID-19 pandemic in sub-Saharan Africa (SSA), according to recent research (Langsi et al., 2021; Coetzee et al., 2022). A web-based cross-sectional study conducted during the lockdown period in most SSA countries found that over half (52.2%) of the participants reported experiencing mental health symptoms, with feeling bored being the most prevalent symptom reported by 70.5% of respondents, followed by feeling anxious (59.1%), being worried (57.5%), frustrated (51.5%), and angry (22.3%; Langsi et al., 2021). The impact of boredom on mental health and emotional responses has been observed across different age groups. A study on adolescents in South Africa found that individuals with higher trait boredom were more likely to use substances (Weybright et al., 2015). Similarly, boredom has also been shown to trigger psychological distress among students in 22 African countries especially during the pandemic lockdown (Zahrae Afellat and Alipour, 2021). The findings of these studies highlight the importance of addressing boredom as a significant mental health concern in Africa. Reduction of adolescent substance use, promotion of leisure/recreation opportunities, and provision of social interaction for children are important strategies to mitigate the impact of boredom on mental health (Weybright et al., 2015; Semo and Frissa, 2020).

## Conclusion

In conclusion, boredom is not a trivial experience, but rather a complex psychological phenomenon that can impact mental health, cognition, and behavior. It is linked to depression, anxiety, substance use, impulsivity, and increased risk-taking behavior. Recognizing and addressing the psychiatric aspects of boredom can play a significant role in promoting mental health and wellbeing. There is a need for research on how to quantify boredom, conduct both quantitative and qualitative research to clearly understand its epidemiological patterns and potential avenues of intervention.

### Strength and limitation

While there is general knowledge that boredom can have both negative and positive effects as well as have significant psychological and psychiatric aspects on individuals, in the African context it can be experienced differently due to the cultural norms, values, and social structures that exist in Africa unlike in other non-African countries. Therefore, in attempt to write about boredom in African setting, it has downed on us on the importance to study boredom in Africa and more so in the Kenyan where we have no documented clinical cases to draw from.

## Author contributions

All authors listed have made a substantial, direct, and intellectual contribution to the work and approved it for publication.
